# The shrimp nephrocomplex serves as a major portal of pathogen entry and is involved in the molting process

**DOI:** 10.1073/pnas.2013518117

**Published:** 2020-10-23

**Authors:** Gaëtan M. A. De Gryse, Thuong Van Khuong, Benedicte Descamps, Wim Van Den Broeck, Christian Vanhove, Pieter Cornillie, Patrick Sorgeloos, Peter Bossier, Hans J. Nauwynck

**Affiliations:** ^a^Department of Virology, Parasitology and Immunology, Faculty of Veterinary Medicine, Ghent University, 9820 Merelbeke, Belgium;; ^b^Center for Environment and Disease Monitoring in Aquaculture, Research Institute for Aquaculture No 1, 222260 Bắc Ninh, Viet Nam;; ^c^Department of Electronics and Information Systems, Faculty of Engineering and Architecture, Ghent University, 9000 Ghent, Belgium;; ^d^Department of Morphology, Faculty of Veterinary Medicine, Ghent University, 9820 Merelbeke, Belgium;; ^e^Department of Animal Sciences and Aquatic Ecology, Faculty of Bioscience Engineering, Ghent University, 9000 Ghent, Belgium

**Keywords:** shrimp, antennal gland, morphology, white spot syndrome virus, *Vibrio*

## Abstract

Viruses, such as white spot syndrome virus, and bacteria, such as *Vibrio* species, wreak havoc in shrimp aquaculture [C. M. Escobedo-Bonilla et al., *J. Fish. Dis.* 31, 1–18 (2008)]. As the main portal of entry for shrimp-related pathogens remain unclear, infectious diseases are difficult to prevent and control. Because the cuticle is a strong pathogen barrier, regions lacking cuticular lining, such as the shrimp’s excretory organ, “the antennal gland,” are major candidate entry portals [M. Corteel et al., *Vet. Microbiol.* 137, 209–216 (2009)]. The antennal gland, up until now morphologically underexplored, is studied using several imaging techniques. Using histology-based three-dimensional technology, we demonstrate that the antennal gland resembles a kidney, connected to a urinary bladder with a nephropore (exit opening) and a complex of diverticula, spread throughout the cephalothorax. Micromagnetic resonance imaging of live shrimp not only confirms the histology-based model, but also indicates that the filling of the diverticula is linked to the molting cycle and possibly involved therein. Based on function and complexity, we propose to rename the antennal gland as the “nephrocomplex.” By an intrabladder inoculation, we showed high susceptibility of this nephrocomplex to both white spot syndrome virus and *Vibrio* infection compared to peroral inoculation. An induced drop in salinity allowed the virus to enter the nephrocomplex in a natural way and caused a general infection followed by death; fluorescent beads were used to demonstrate that particles may indeed enter through the nephropore. These findings pave the way for oriented disease control in shrimp.

## Significance

The ever-growing human population faces problems providing sufficient animal proteins. For shellfish, wild catch is supplemented by aquaculture to meet this increasing global demand. However, aquaculture faces serious problems with infectious diseases. One of the main problems for oriented disease control is lack of information on pathogen entry in the host. The present study fully describes the anatomy of the shrimp’s excretory organ, the antennal gland, identifying the organ as a major portal candidate. Additional findings show that pathogens may indeed enter through this organ naturally, infecting shrimp. We also demonstrate involvement in molting. These insights into the molting process and pathogen entry open doors in fundamental biology and the potential development of disease control measures.

White spot syndrome virus (WSSV) is regarded as a major cause of production losses within crustacean aquaculture, which is estimated at 10% or several hundred million US$ annually (1–3). This virus is of particular concern to leading scientists because it hinders the future global food supply, as aquaculture is one of the most important food sources to meet the increasing demand of a growing global population (4). Viruses are not the only pathogens known to cause serious damage in shrimp industry; bacterial infections such as vibriosis cause an estimated 20% production loss annually (5).

Three routes have been proposed in the past for the transmission of WSSV: 1) through feeding of infected shrimp (6, 7); 2) waterborne, by exposure to water contaminated with WSSV (8–10); and 3) transovarially (11). The gut (per os) has been speculated to be the primary target organ that allows WSSV to enter shrimp. However, the efficiency with which infection can be induced via this route is extremely low and remains controversial (6, 12, 13). Consequently, there is still a lot of debate on the exact portal of entry (10, 14–16). The gut, along with almost every other structure, is completely covered by a nonpenetrable layer of cuticula or a peritrophic membrane (12, 17). Recent research showed that the cuticula and peritrophic membrane indeed serve as firm barriers against WSSV (12, 15). Therefore, the question was raised on how WSSV (and other pathogens such as *Vibrio*) can overcome this barrier. In our search for structures that are connected to the outside world but have no cuticle-lined lumen, we came across the antennal gland. Therefore, we opted to investigate the potential of the antennal gland as a portal of entry for pathogens. The antennal gland has been reported to be among the very first organs to become infected, using immunohistochemistry (18). Furthermore, Thuong et al. (19) showed increased susceptibility to WSSV infection upon sudden change of salinity. Hemolymph filtration and osmoregulation is the primary function of the antennal gland (20, 21); therefore, these findings further strengthened our suspicion in the direction of the antennal gland as a major portal of pathogen entry.

Previous endeavors have been undertaken to chart the morphology of the antennal gland. The generally accepted structure of the shrimp’s bilateral excretory organ can be described as a three-part organ: 1) a coelomosac, responsible for filtration of the hemolymph; 2) an efferent labyrinth which alters the filtrate; and 3) a terminal ductus consisting of a bladder and a small ductus leading to the nephropore. This nephropore seals the antennal gland from the outside world (20, 22, 23). After injection of a colored fluid, Young (23) found traces of this fluid around the supraesophageal ganglion (the brain) of the shrimp. Finally, two Japanese studies made an incomplete clay model of the shrimp’s antennal gland, suggesting a much larger configuration of the coelomosac (24, 25).

Antecedently, some pathogens have been retrieved from the antennal gland of Crustacea. Thrupp et al. (26), for example, found a parasitic infection in the antennal gland of juvenile crabs. In the same manner, Pina et al. (27) isolated *Cercaria sevillana* cysts from the green crabs antennal gland, whereas other researchers found a herpesvirus-like particle through electron microscopical examination of the antennal gland (28). Thus, the presence of pathogens inside the antennal gland is not a new given. However, to this day, no evidence has been provided that the antennal gland serves as an entry portal for pathogens and can act as a primary replication site. The current study aims to investigate the role of the antennal gland as a possible entry route and primary replication site of WSSV and *Vibrio* species.

## Results

### The Antennal Gland’s Complexity Unveiled.

Upon dissection of the antennal peduncle, a clean, white, bean-shaped structure becomes visible. This structure is bilaterally present and located at the transition from peduncle to cephalothorax and is the compact glandular compartment (CGC) of the antennal gland. This connects to a urinary bladder, only visible on a crosscut of the unopened hemocoel. Depending on the size of the animal, this urinary bladder can be visualized either by bright-field microscopy or the naked eye.

**Fig. 1.**
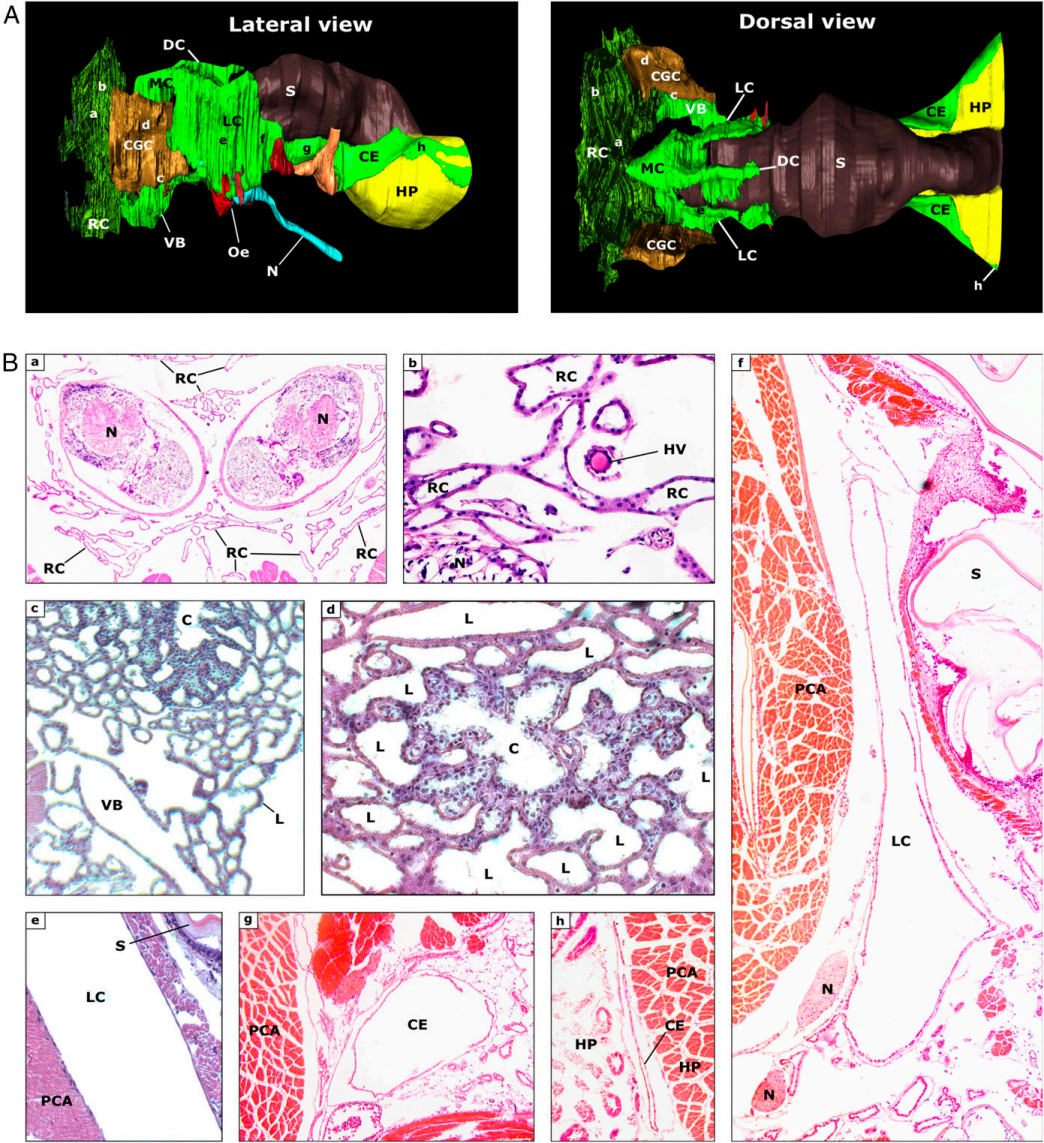
Morphology of the nephrocomplex and surrounding areas. (*A*) A 3D reconstruction of the nephrocomplex by superposition of serial hematoxylin and eosin-stained cross-sections of the cephalothorax. (*B*) Relevant cross-sections of important components of the nephrocomplex at levels a through h, as indicated in Fig. 1*A*. C, coelomosac; CE, caudal extension; DC, dorsal lobe of the MC; HP, hepatopancreas; HV, hemolymph vessel; L, labyrinth; MC, median compartment/diverticulum; N, nerve; Oe, esophagus; PCA, protocephalon attractor; RC, rostral compartment; S, stomach; VB, ventral bladder.

Three-dimensional (3D) reconstruction of the antennal gland and surrounding organs by superposition of serial hematoxylin and eosin-stained sections (10 µm) of the cephalothorax revealed a much more complex and widespread organization of this organ than previously believed (Fig. 1*A*; 3D-rendering as Movie S1). The entire antennal gland can be divided into several compartments. The macroscopically visible CGC is both histologically and functionally subdividable (Fig. 1*B*). Centrally, the hemolymph-filtering coelomosac can be found, surrounded by the filtrate-modifying tubuli, collectively known as the labyrinth. The coelomosac comprises a central lumen encompassed by podocytes with big, irregular nuclei, and little cytoplasm. The antennal artery penetrates the CGC. The coelomosac will eventually branch out to form the tubuli of the labyrinth. These tubuli bathe in the hemolymph, and their cells are cubical to columnar. They possess a brush border and a basally located nucleus. We found that the tubuli of the CGC spread out in the rostral direction, gradually filling the entire cephalon, surrounding the supraesophageal ganglion. Here, both sides of the labyrinth end blindly and are connected with each other, thus forming one rostral compartment of the antennal gland.

**Fig. 2.**
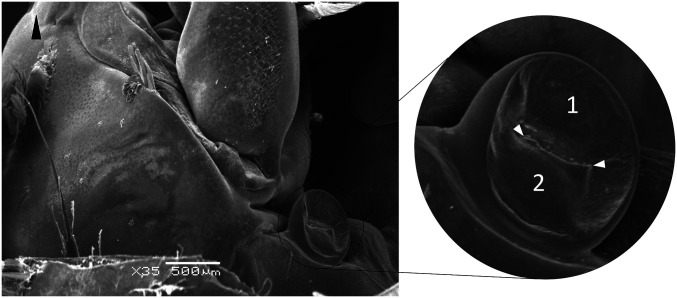
SEM image of the nephropore at the antennal base of *P. vannamei* shrimp. Black arrowhead is pointing to the rostrum; 1, rostral valve; 2, caudal valve; white arrowheads, fissure.

The tubuli of the rostral compartment eventually return to the CGC, where they end up in the ventral urinary bladder. Contrary to most other parts of the antennal gland, the bladder wall is made up of multiple cell layers. The epithelium is a very thin layer, characterized by protruding nuclei. Just like the labyrinth, the bladder epithelial cells also possess a brush border. Thereunder, layers resembling smooth muscle and connective tissue can be found. The urinary bladder is positioned between three important muscles: medially, the coxopodite adductor and the minor scaphocerite abductor; ventrally, the major scaphocerite abductor. The bladder has two major extensions in addition to some ramifications of minor importance. The first extension is a short, narrow, terminal duct leading to the nephropore. The transition between the ventral bladder and this terminal duct is jammed between the coxopodite adductor muscle (dorsal) and the large scaphocerite abductor muscle (lateroventral). The second extension of the ventral bladder leads deeper into the cephalothorax, where the two ventral urinary bladders connect right in front of the esophagus, forming the preesophageal connection (PEC). Scanning electron microscopy (SEM) images of the nephropore, revealed a valve-like closing of the pore (Fig. 2). The rostral valve is superimposed by the caudal valve, forming a check valve configuration.

**Fig. 3.**
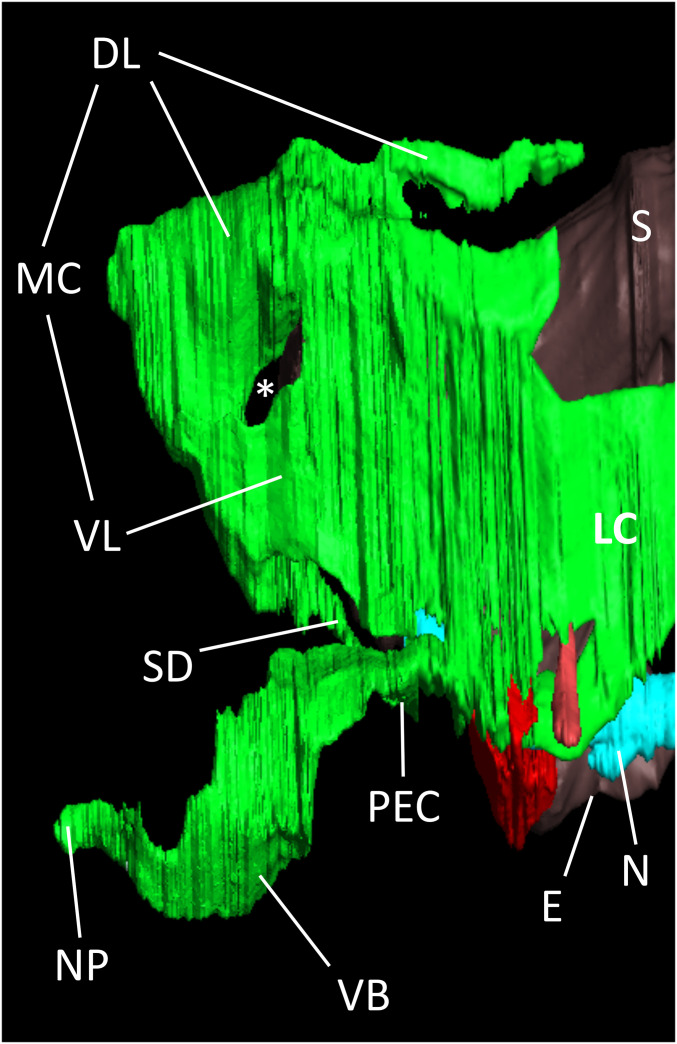
Lateral view of the vesicular structures of the antennal gland without the rostral compartment and the compact glandular part. S, stomach; E, esophagus; N, nervus; NP, nephropore; VB, ventral bladder; MC, median compartment, VL, ventral lobe of the median compartment; DL, dorsal lobe of the median compartment; SD, square duct; *, space for the large protocephalon attractor muscle.

From the PEC, two lateral diverticula rise, designated as the lateral compartments (LCs) (Fig. 3). These are positioned between the esophagus and the proventriculus medially and the large protocephalon attractors laterally. Here, the LC has only one flat cell layer and is attached to the two adjacent structures. When followed in the caudal direction, the LC loses contact with the lateral muscle and medial stomach, decreases in height, and eventually runs along the ventral side of the alimentary tract as two caudal extensions. These extensions end blindly with a connection rostrally from the hepatopancreas. The LC’s caudal extensions only possess a single cell layer. Along the path of these caudal extensions, the structure regularly encounters the following muscular structures: 1) the maxillary tensors, 2) two previously undescribed small oblique muscles (oriented like the cruciate ligaments in the mammalian knee), and 3) the mandibular adductors. These structures are, on one side, connected to the stomach and, on the other side, attached to the exoskeleton, trapping the caudal extensions between the two.

The two LCs are connected to each other rostrally from the esophagus and stomach. This connection has a wide volume and was named the median compartment (MC). A muscular layer topped off by a flattened epithelium makes up its cellular structure, indicating a dynamic structure. The median compartment can be subdivided into a ventral lobe and a dorsal lobe. The latter runs dorsally from the stomach and ends there blindly. The ventral lobe of the median compartment is also connected to the PEC through a square duct. Again, several muscles were found in association with this part of the antennal gland. The combined effort of the large protocephalon attractor muscle, the epistomal stator muscle, and the gastric mill muscle may squeeze the ventral lobe and frontal part of the dorsal lobe, moving its content into the posterior part of the dorsal lobe.

**Fig. 4.**
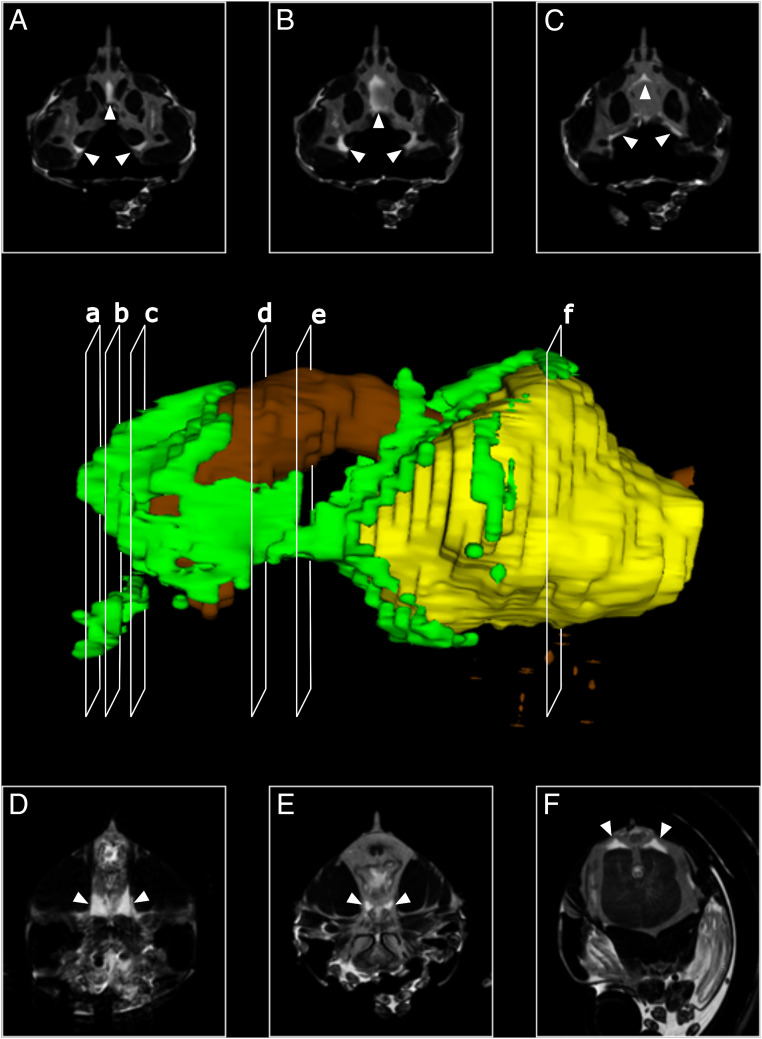
A 3D reconstruction of the nephrocomplex based on cross-sections with µMRI. Green, vesicular compartments of the nephrocomplex; brown, intestinal tract; yellow, hepatopancreas. (*A*–*F*) µMRI images representing cross-sections of the 3D model. Arrows indicate parts of the nephrocomplex.

Besides the histological 3D reconstruction with AMIRA software V6.0, we applied a different imaging technique to confirm the correctness of the histology-based 3D reconstruction: serial micromagnetic resonance imaging (µMRI). Because of the strong signal correlated with water in the animal, this technique allowed us to confirm the in vivo structure of the antennal gland (Fig. 4).

The ventral urinary bladder, PEC, median compartment, LC, and the caudal extensions as revealed by the histological 3D reconstruction were clearly distinguishable on the µMRI images, as the contents gave a stronger signal compared to the surrounding tissues, resulting in sharp contrast. However, the CGC and the rostral compartment were not visible on µMRI.

**Fig. 5.**
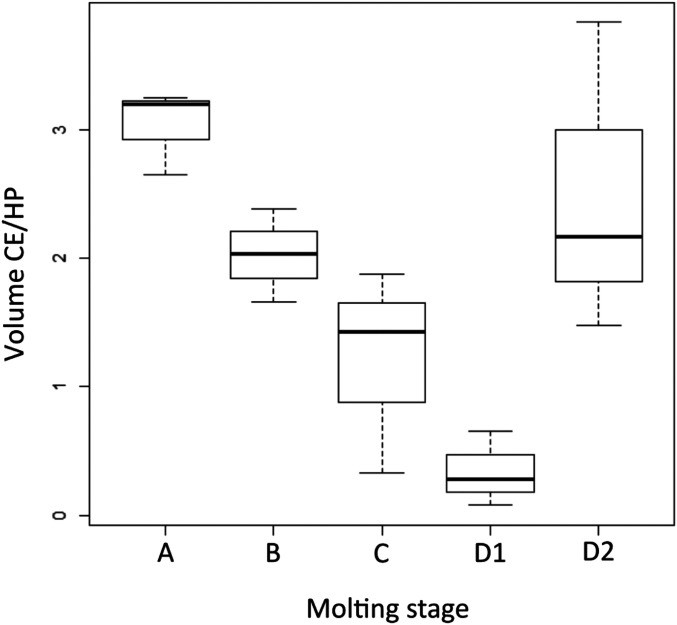
Box plot representing the ratio of the volume of caudal extensions of the antennal gland (CE) over the volume of the hepatopancreas (HP) on µMRI. The box plots show the changes of the volume ratio over the different molting stages.

The µMRI revealed an additional finding: Namely, the filling of the caudal extensions seemed to be linked to the molting stage of the shrimp (Fig. 5). The contents volume gradually decreased according to the progressing molting stage to reach a minimum in D1 (early premolt). In D2 (late premolt), the contents increase substantially. Around ecdysis (stages D2 and A), the volume of the caudal part is significantly greater compared to the volume observed in animals that were in intermolt (stages B, C, and D1). The volume of the hepatopancreas (also visible on the µMRI) was used as a reference point because the size of this organ does not vary over different molting stages. The contents of the caudal extensions of the antennal gland in perimolt were, on average, 3.25 times greater when compared to intermolt.

### Infection upon Intrabladder Inoculation.

The results obtained from the 3D reconstruction and SEM helped with the design of an inoculation method: the intrabladder inoculation. Hereby, a catheter of 26G was inserted in the terminal duct of the ventral urinary bladder, and a liquid was injected. The method was validated using contrast X-ray microcomputed tomography. Up to 100 µL of contrast fluid can be inserted into a shrimp of 25 g without bursting the nephrocomplex. We compared the infectivity of WSSV and *Vibrio campbellii* after intrabladder inoculation with peroral and intramuscular inoculations (29). The experiment was performed three times (*SI Appendix*, Tables S1 and S2).

For a given WSSV Thai-1 stock, an infectious titer of 10^8.67^, 10^8.67^, and 10^8.80^ shrimp infectious dose 50% end point (SID_50_) mL^−1^ (x̅ = 10^8.71^ SID_50_ mL^−1^) was achieved after intramuscular inoculation; 10^1.14^, 10^1.30^, and 10^1.30^ SID_50_ mL^−1^ (x̅ = 10^1.25^ SID_50_ mL^−1^) upon peroral inoculation, and 10^6.97^, 10^6.60^, and 10^7.30^ SID_50_ mL^−1^ (x̅ = 10^6.96^ SID_50_ mL^−1^) after intrabladder inoculation. All dead and moribund shrimp were WSSV positive, while surviving shrimp were WSSV negative. Taken together, this means that, compared with the intramuscular route, only 56 times more infectious virus is needed to infect shrimp via intrabladder inoculation, whereas 28.8 × 10^6^ times more virus is necessary to infect shrimp via oral inoculation.

For a given *V. campbellii* stock, a similar experiment was performed: 10^4.16^, 10^4.37^, and 10^4.16^ lethal dose with 50% end point (LD_50_) mL^−1^ (x̅ = 10^4.23^ LD_50_ mL^−1^) was found on intramuscular inoculation, and 10^2.50^, 10^2.50^, and 10^2.32^ LD_50_ mL^−1^ (x̅ = 10^2.44^ LD_50_ mL^−1^) on intrabladder inoculation. It was not possible to kill shrimp using the peroral route. Determination of bacteria by plate counting indicated that all dead and moribund shrimp were *V. campbellii* positive and contained high densities of *V. campbellii* (3.8 ± 1.0 × 10^5^ colony forming units [cfu] mL^−1^ of homogenate), whereas surviving shrimp showed a clearing mechanism to eliminate bacteria from their body (2.4 ± 3.4 × 10^2^ cfu mL^−1^ of homogenate). Taking all results together, these findings demonstrate that, compared with the intramuscular route, only 62 times more *V. campbellii* is required to kill shrimp via intrabladder inoculation, whereas it was not possible to kill shrimp by the peroral route with up to 10^9.0^ *V. campbellii.*

Next, the pathogenesis of a WSSV infection in this organ was examined after intrabladder inoculation. Intrabladderly and intramuscularly inoculated animals were compared (*SI Appendix*, Fig. S3 and Table S4). In the intramuscular inoculation group, the first WSSV-infected cells were observed at 18 h postinoculation (hpi) in all investigated tissues: ventral urinary bladder, CGC (coelomosac and labyrinth), heart, gills, lymphoid organs, hepatopancreas, hematopoietic tissues, and cuticular epithelium of head, body, and hindgut. WSSV-positive cells in hemolymph were detected at 24 hpi. Upon intrabladder inoculation, WSSV-infected cells were only detected in the epithelial cells of the bladder at 18 hpi. From 24 hpi onward, WSSV-positive cells were seen in the other investigated tissues and in some cells in the hemolymph. With both inoculation routes, only a small proportion (<2%) of cells in the hemolymph were WSSV positive. WSSV-infected cell numbers of all investigated tissues were low at 24 hpi. The number of WSSV-infected cells then increased rapidly and reached high levels after 36 hpi. The highest count of WSSV-infected cells was found in the coelomosac, labyrinth, gills, and the cuticular epithelial cells. The number of WSSV-infected cells observed in the intramuscular inoculated shrimp was higher than that observed in the intrabladder inoculated animals in all investigated tissues.

### Natural Infection via the Nephrocomplex.

**Fig. 6.**
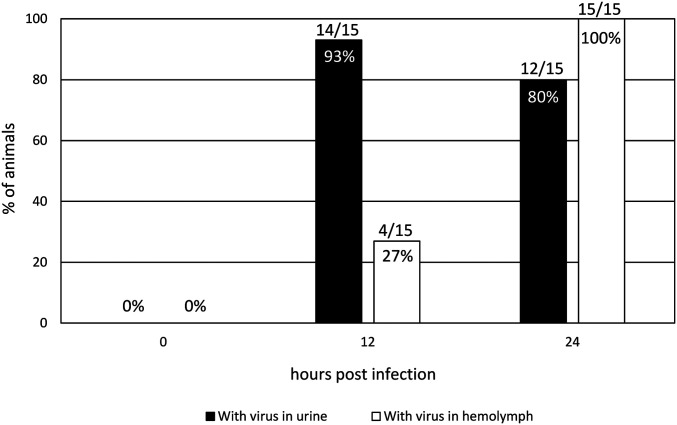
Presence of WSSV in urine and hemolymph before and 12 and 24 h after immersion inoculation during a salinity drop.

The intrabladder inoculation showed that the antennal gland is efficiently infectable by WSSV and *V. campbellii*. To reproduce a natural infection, we exposed shrimp to WSSV (10^5.5^ SID_50_ mL^−1^; 1 L) during a drop in salinity (from 35 g⋅L^−1^ to 5 g⋅L^−1^) for 5 h and analyzed the outcome (Fig. 6 and *SI Appendix*, Table S5; three experiments with two controls). Urine and hemolymph samples were taken every 12 h. During a hyposalinity exposure, shrimp try to maintain their hemolymph osmolarity by expelling fluid via their excretory system, which leads to more frequent urination (30). The frequent opening of the nephropore may lead to pathogen exposure. The virus could be detected with a qPCR in the urine 12 h postinfection, while the hemolymph mostly stayed negative. Only after 24 h, the virus could be detected in the hemolymph of all but one shrimp. Increasing amounts of WSSV copies were found in the urine and hemolymph at later time points. All shrimp were dead 48 hpi. Shrimp of control group 1 (drop in salinity; no WSSV) and of control group 2 (no salinity drop; with WSSV) remained completely virus negative, and all shrimp survived.

The SEM (Fig. 3) revealed a check valve-like barrier function of the nephropore. An ex vivo model was set up to test whether a buildup in urine pressure on the inside of the valve is sufficient for fluid evacuation (simulating the mictio). By dissecting the shrimp and carefully removing all tissue inside the antennal appendix, we were able to mount the nephropore on the tip of a 50-mL syringe. Fixation was performed with superglue and TEC7. Water was poured into the syringe until drops started to leak out of the nephropore. The height of the water level in the syringe (mmH_2_0) when fluid started leaking out of the nephropore, corresponds to the hydrostatic pressure, and this was found to be equal over different molting stages (2.8 bar; *SI Appendix*, Table S6). When the nephropore was reversely mounted on the syringe tip, no leakage was found even after high manual pressure was exerted (simulating water pressure from outside to inside of the shrimp). This confirms the barrier function exerted by the check valve-like nephropore configuration at rest.

During a salinity shock, the antennal gland functions as a volume regulator, resulting in an increased mictio frequency when the ambient salinity drops below the salt concentration inside the shrimp. To find out whether the urination process compromises the barrier function of the nephropore valves, another ex vivo model was designed. The nephropore was mounted on the tip of a syringe filled with water. Mictio was simulated with the nephropore bathing in a 35‰ salt solution containing green fluorescent particles (0.2 and 1 µm, representing WSSV and *V. campbellii*, respectively) by emptying the syringe (pushing the plunger) and abruptly retracting the plunger (creating a vacuum inside the syringe). After the simulation, these particles were found inside the syringe by confocal microscopy, demonstrating that particles easily cross the barrier model.

## Discussion

In this study, we explored the anatomy of the antennal gland in full. Besides the already known structures (17), that is, nephropore, terminal duct with urinary bladder, labyrinth, and coelomosac, the latter two forming the CGC, we have additionally uncovered a rostral continuation of the labyrinth surrounding the supraesophageal ganglion, coined as the rostral compartment. The ventral urinary bladder appears to possess several continuations: 1) a terminal duct leading to the nephropore and ultimately to the outside world; 2) the PEC connecting the contralateral urinary bladder; and 3) the lateral compartments (LCs), two bilateral large sheets flanking the stomach. These LCs flow into the caudal extensions, ending blindly at the height of the hepatopancreas. Rostrally from the stomach, the LCs are connected with each other, forming a median compartment. The two large protocephalon attractor muscles subdivide the median compartment into a ventral and a dorsal lobe.

In 1959, Young (23) described colorations above and below the supraesophageal ganglion after introducing dye in the nephropore using *Penaeus setiferus* as subject. We are convinced these colorations are nothing other than the rostral compartment (23). We also theorize that the rostral compartment might be homologous to the loops of Henle, found in mammalian kidneys. Nakamura and Nishigaki (25) also described a larger and more complex anatomy, using a clay model in *Penaeus japonicus*. However, they stated that there is only one continuation of the ventral urinary bladder, the coelomosac. Based on the histology, we disproved this theory. Furthermore, many of the structures described in their paper are different or have an altered configuration in comparison to this study performed in *Penaeus vannamei* (24, 25).

The ventral urinary bladder is surrounded by three major muscles: medially, the coxopodite adductor and the minor scaphocerite abductor; ventrally, the major scaphocerite abductor. These muscles presumably play a major role in squeezing the ventral urinary bladder to empty its contents, aided by the smooth muscle layers residing in the bladder wall. The transition between the ventral bladder and this terminal duct is jammed between the coxopodite adductor (dorsal) and the large scaphocerite abductor (lateroventral). No other muscular structure was found capable of performing a sphincter-like function. Therefore, we assume that the urinary bladder is sealed by a combination of the two latter muscles and the two overlying layers of the exoskeleton (the valves of the nephropore). This means that the pressure inside the ventral bladder should be increased in order to open the valves of the nephropore by using a coordinated contraction and/or relaxation of the muscles described above.

The quality of the histology-based 3D model may seem under par but is due to the presence of a thick cuticular shield (the carapax) which makes slicing ultrathin cross-sections (<10 µm) extremely difficult. We opted not to remove this carapax, because it may have altered the localization and orientation of some extremely thin structures of interest. We noticed that, on subsequent slices, the contents were not always perfectly aligned, leading to lower 3D quality. We theorize that the pressure of the knife on the carapax may have induced minor changes to the underlaying tissues, slightly mismatching the alignment of serial sections.

Never before was the internal anatomy of shrimp studied using µMRI. The experiment proved to be successful, as the signal from the water content of the larger parts of the organ was clearly distinguishable from that of the hemolymph. Overall, the antennal gland followed the same blueprint as revealed by the histology-based AMIRA reconstruction, thus, confirming the histology-based 3D reconstruction in vivo. Only the rostral compartment and the CGC were not visible on µMRI. Additionally, the µMRI reconstruction revealed the caudal extensions running dorsally and ventrally of the hepatopancreas in contrast to the histology-based model, where the caudal extensions run laterally from the hepatopancreas. Moreover, the dynamic change of the contents of the caudal extensions during the different stages of molting might indicate a functional role in the molting process. Given the higher volume and the dorsal location of the extensions as seen on the µMRI before molting, we theorize that the fluid filling of the antennal gland contributes to the rise of the pressure on the dorsal inner side of the carapax to help tear the ligament between the cephalotohorax and the abdomen during ecdysis. Along the caudal extensions, the maxillary tensors, the two cruciate oblique ligaments, and the mandibular adductors possibly act in a coordinated fashion, pumping fluid to the end of the caudal extension. Shortly after molting, the volume of the caudal extensions remains high, helping to increase the volume of the shrimp as much as possible, while the exoskeleton hardens after molting. Afterward, the volume taken up by fluids in the caudal extensions could progressively be replaced by tissue, explaining the relapse of the volume of the caudal extensions over progressing molting stages. This combined effort would allow the shrimp to increase its body size over consecutive molting cycles. Selection on the size of the inner antennal gland diverticles may lead to faster-growing shrimp.

In conclusion, the antennal gland has a substantially wider distribution throughout the cephalothorax and is a much more complex structure than previously assumed. The unraveling of the complete structure indicates that the antennal gland is a perfect portal for pathogen entry and that its wide distribution in the shrimp cephalothorax results in close contact to all WSSV-susceptible organs: the nervous system, the alimentary tract, the lymphoid organ, the gills, the hepatopancreas, and various muscles. Only the heart is not in close contact with the antennal gland. Most of the antennal gland’s cell layers are singular and have no cuticular lining, which allows for a potential rapid entry of pathogens into the hemolymph. Because of the vast distribution of the organ in the cephalothorax, not limited to the antennal peduncle, the antennal gland’s function as an excretory organ, and the complex of diverticles in connection with the bladders, we propose a name for this organ: the nephrocomplex. The prefix “nephron” is from the Greek “*nephros*,” for kidney, and the suffix “complex” is because of the large numbers of diverse subunits that the excretory organ consists of.

The hypothesis of the nephrocomplex as a major pathogen entry portal candidate was confirmed by intrabladder inoculation of shrimp with WSSV and *V. campbellii*, leading to both morbidity and mortality. We demonstrated that these pathogens could easily infect shrimp through inoculation of the nephropore (intrabladder). Compared to intramuscular inoculation, only 56 times more infectious WSSV is needed to infect shrimp via intrabladder inoculation, while 28.8 × 10^6^ times more virus is necessary to infect shrimp via oral inoculation. For *Vibrio*, similar results were obtained (62 and >10^9.0^ times more, respectively). These data show that the intrabladder infection is almost as efficient as the intramuscular inoculation, which bypasses all natural defense barriers such as nephropore valves, cuticula, and peritrophic membrane. In contrast, the peroral route was significantly less efficient. This was in accordance with the findings of Thuong et al. (12). In the pathogenesis experiment, we clearly demonstrated that, when infected via the nephropore, WSSV first replicates in the epithelial cells of the bladder at 18 hpi and, afterward, spreads all over the body. This proves the possibility and efficiency of viral spread from the tissues of the nephrocomplex into the hemocoel, where the virus can infect all other susceptible organs.

Intrabladder inoculation proved to be successful in infecting shrimp with pathogens. Nevertheless, introducing the pathogens directly into the target organ, thereby avoiding natural barriers, is very artificial. A drop in salinity has been proven to facilitate WSSV infection (19). We found that, during such conditions, shrimp urine tested WSSV qPCR positive before hemolymph. After 24 hpi and farther on, the presence of increasing amounts of WSSV copies further supports the nephrocomplex’s role as primary replication site. However, to provide strong evidence that the nephrocomplex functions as a natural portal of entry, proof of pathogen entry was required. SEM images of the nephropore suggested a valve-like closing of the pore, where the caudal valve is superimposed on the cranial, assuring an effective check valve sealing of the nephrocomplex. The integrity of this nephropore barrier function was tested using an ex vivo setup, where pressure from inside of the shrimp (simulated mictio) allowed the valves to open, whereas pressure from outside of the shrimp did not cause the valve seal to be compromised. This indicated that, in a closed position, water cannot enter the shrimp, and thus the cuticular barrier remains impenetrable for pathogens. Under normal conditions, the only time this cuticular valve opens is during urination. Specific conditions during which the nephropore opens more often could provide pathogens a window of opportunity for pathogen invasion. A sudden drop in salinity, resulting in frequent urination (and thus frequent opening of the nephropore), is such a condition. Because of the nephrocomplex’s role in regulation of hemocoel volume, the sudden decrease in salinity prompts the shrimp to produce and expel urine in higher quantities and at higher frequency (30). During this release of urine through the nephropore, the valves are open. We hypothesize that, right between the end of the urinal efflux and the closure of the valve, there is a short timeframe during which there may be an influx of external water with contaminating pathogens into the nephrocomplex. When the pathogens are highly virulent, little virus is needed to establish an infection, as proven by the inoculation trials. With another ex vivo model setup, mictio was simulated, and the crossing of particles with the size of WSSV (200 nm) and *Vibrio* (1 µm) at the end of the urination process was demonstrated. All these findings together provide strong evidence for the nephrocomplex to be a major portal of pathogen entry.

## Conclusion

The nephrocomplex, previously known as the antennal gland, is revealed to be much more complex than previously assumed. Its anatomy, morphology, and cellular structure are optimal for the excretion organ to be a pathogen entry portal. Also, links to the molting process were found using µMRI. Additionally, the sealing function of the nephropore valves was examined and found to be an efficient pathogen barrier. However, it was demonstrated that, at the end of the urination process, this function is briefly compromised. Thus, during conditions where frequent urination takes place (sudden salinity drop during, e.g., heavy monsoon rains, aggression, establishment of social dominance, and feed intake) combined with a high virus titer in the surrounding water, the nephrocomplex has to be considered a major portal of pathogen entry (10, 31, 32).

The findings in this paper will cause a major shift in shrimp pathogen research, especially in the field of WSSV where all current findings are, until now, solely based on intramuscular and peroral inoculations. WSSV pathogenesis and immunity studies have to be performed using intrabladder inoculation or via immersion upon drop in salinity. Also, the identification of the nephrocomplex as an entry portal will focus the search for control measures to this organ. It will also allow for a direct breeding program for pathogen resistance. Finally, it confirms the empirical observation of shrimp farmers that periods of heavy rainfall are linked to major outbreaks of WSSV infections in open-air ponds.

## Materials and Methods

### Infectivity Study.

#### Experimental animals.

Specific pathogen-free (SPF) penaeid shrimp, *P. vannamei*, with a mean body weight (MBW) of 2.8 g were reared in the Aquaculture & Artemia Reference Center (ARC), Ghent University. Shrimp were cultured in a biofilter circulation system, fed with pelleted feed at a rate of 5% of their mean body weight per day. Temperature and salinity of the culture system were maintained at 27 ± 1 °C and 35 ± 1 g⋅L^−1^. Total ammonia and nitrite were controlled to be lower than 0.5 and 0.15 mg⋅L^−1^, respectively. For the experiments, shrimp in early premolt were screened (15) and transported to the Laboratory of Virology, Faculty of Veterinary Medicine, Ghent University.

#### WSSV production.

The WSSV Thai-1 used in the present study was collected from naturally infected *Penaeus monodon* in Thailand in 1996 and passaged in crayfish *Pacifastacus leniusculus* (33). Crayfish gill suspension containing WSSV Thai-1 was kindly donated by K. Söderhäll, Uppsala University, Uppsala, Sweden. The virus was amplified in SPF *P. vannamei* juveniles to produce a virus stock. The median infectious titer of the stock was 10^6.6^ SID_50_ mL^−1^ i.e., 10^6.6^ times the experimentally determined dose of virus needed to infect 50% of shrimp, as determined by in vivo intramuscular titration in SPF *P. vannamei* (17). A 10^−2^ dilution of this stock was made in phosphate-buffered saline (PBS), pH 7.4, and injected intramuscularly into SPF *P. vannamei* juveniles to amplify the virus. Then, moribund shrimp were collected and confirmed to be WSSV positive by indirect immunofluorescence (IIF). Thawed shrimp without shell, hepatopancreas, and gut were chopped, suspended in PBS at a ratio of 1:3, homogenized at 5,000 rpm for 1 min using an IKA T 25 digital Ultra-turrax, and centrifuged at 5,000 × *g* for 20 min (4 °C). Supernatant was collected, filtered (0.45 µm), and aliquoted for storage at −70 °C. All manipulations were done inside a laminar flow cabinet under sterile and precooled conditions.

#### *V. campbellii* production.

Rifampicin-resistant bacteria (*V. campbellii*: LMG21363) that are pathogenic for penaeid shrimp were obtained from the ARC, Ghent University. From the original stock, 20 µL of bacterial suspension was inoculated in 20 mL of Marine Broth 2216 (Difco Laboratories) containing 100 mg⋅L^−1^ of rifampicin for 12 h at 27 °C in a shaker at 90 rpm. Then, the bacteria were subcultured under the same conditions for 14 h. Afterward, the suspension was washed and centrifuged three times at 2,000 × *g* for 10 min. Finally, an estimated concentration of 10^10^ cfu mL^−1^ of bacterial suspension (stock) was made by determining the optical density using spectrophotometry at an absorbance of 600 nm (OD_600_). An optical density value (OD_600_) of 1.0 corresponds to 1.2 × 10^9^ cells per mL^−1^ (McFarland standard).

#### Infectivity of WSSV stock in *P. vannamei* by different routes of inoculation.

The aim of this study was to compare the infectivity of WSSV stock in shrimp using different inoculation routes: intramuscular injection, peroral, and intrabladder inoculation. In the experiment, early premolt *P. vannamei* (25.4 ± 3.3 g) were collected and acclimated individually for 24 h in 10-L tanks. Then, 15 shrimp were injected intramuscularly with 5 µL of a 10-fold serial dilution (10^−^5, 10^−6^, 10^−7^) of the WSSV stock, as described in *Materials and Methods*, per animal (five animals per dilution). Twenty shrimp were inoculated perorally with 50 µL of a 10-fold serial dilution (10^0^ to 10^−3^) of the same WSSV stock per animal (five animals per dilution). A further 20 shrimp in the third group were inoculated into the bladder of the antennal gland with 5 µL of a 10-fold serial dilution (10^−3^ to 10^−6^) of the same WSSV stock per animal (five animals per dilution). The inoculation procedures were fully standardized. Briefly, intramuscular injection was performed with a 25-gauge needle (Terumo) mounted on an accurate syringe (Model 1710 LT SYR, 100 µL, Hamilton Bonaduz) filled with 5 µL of WSSV suspension. For oral inoculation, shrimp wrapped in tissue paper were placed ventral side up under a stereomicroscope. The tip (2 mm) of a 0.64 × 19 mm 26G Surflo-W catheter (Terumo) mounted on a 100-µL Hamilton Bonaduz, filled with 50 µL of a WSSV suspension, was introduced into the oral cavity, and the inoculum was delivered into the lumen of the foregut. For intrabladder antennal gland inoculation, first, urine was removed from the bladder after gently introducing the tip (0.5 mm) of a 0.64 × 19 mm 26G Surflo-W catheter (Terumo) in the nephropores. The catheter was kept stable for a few seconds until the urine fully filled the catheter. Afterward, the catheter was removed and replaced with a new 0.64 × 19 mm 26G Surflo-W catheter (Terumo) connected with a 100-µL Hamilton Bonaduz syringe, filled with 5 µL of a WSSV suspension. The WSSV suspension was inoculated by exerting a gentle pressure on the plunger of the syringe. After inoculation, shrimp were housed individually and kept for 5 d. Cephalothoraxes of dead and moribund shrimp were collected every 12 h. The experiment was terminated at 120 hpi. At the latter time point, all surviving shrimp were humanely killed. Samples of dead, moribund, and killed shrimp were processed by IIF for detection of WSSV-infected cells. The experiment was performed in triplicate.

#### Lethality of *V. campbellii* in *P. vannamei* by different inoculation routes.

From a bacterial stock (10^10^ cfu mL^−1^), serial dilutions (10^−^1, 10^−^2, 10^−^3, 10^−^4) were made in filtered, autoclaved seawater (FASW). Then, these dilutions were used to determine the LD_50_ mL^−1^ in *P. vannamei* by intramuscular injection, peroral, and intrabladder inoculations. Fifteen shrimp were injected with 100 µL of a 10-fold serial dilution (10^−^2, 10^−^3, 10^−^4) of the *Vibrio* stock, as described in *Materials and Methods*, per animal (five animals per dilution). Fifteen shrimp were inoculated perorally with 100 µL of a 10-fold serial dilution (10^0^, 10^−1^, and 10^−2^) of the same *Vibrio* stock per animal (five animals per dilution). Fifteen shrimp in the third group were inoculated intrabladder with 10 µL of a 10-fold serial dilution (10^0^, 10^−1^, and 10^−2^) of the same *Vibrio* stock per animal (five animals per dilution). After inoculation, shrimp were housed individually and kept for 5 d. Dead and moribund shrimp were collected every 6 h, and the experiment was terminated at 120 hpi. At the latter time point, all surviving shrimp were humanely killed. For counting the bacterial density in dead, moribund, and killed shrimp, shrimp were washed once with 70% alcohol and twice with FASW. Then, the whole shrimp were homogenized in FASW at a ratio of 1:5 (10^0^) and diluted (10^−^1, 10^−^2, 10^−^3) in FASW. One hundred microliters of each dilution was plated on marine agar containing 100 mg⋅L^−1^ rifampicin. The plates were incubated at 28 °C for 24 h, then counting was conducted, with each plate containing between 30 and 300 colonies.

#### Replication of WSSV in *P. vannamei* after intramuscular and intrabladder inoculation.

Early premolt SPF shrimp (25.2 ± 3.8 g), acclimated for 1 d at 27 °C, were divided into three groups. Animals were injected intramuscularly with 10 µL containing 10^5.5^ SID_50_ of WSSV, at the junction between the fourth and fifth abdominal segment. Animals were inoculated intrabladder with 10 µL containing 10^5.5^ SID_50_ of WSSV. Animals were mock inoculated by intramuscular injection with 10 µL of PBS and used as controls. After inoculation, blood and cephalothoraxes of five shrimp were sampled at 0, 6, 12, 18, 24, 36, and 48 hpi. For the determination of WSSV-infected cells in hemolymph, 500 µL of hemolymph were collected with anticoagulant buffer at a ratio of 1:1. Hemolymph was further diluted 1:1 in PBS. Then, 100 µL of the diluted hemolymph was cytospinned. The cytospins were centrifuged at 300 × *g* for 4 min, and then fixed for 10 min in methanol. WSSV viral antigens were then detected by IIF. For the determination of WSSV-infected cells in tissues, the cephalothoraxes were cross-sectioned (20 μm) at the site of the antennal gland, hepatopancreas, gills, lymphoid organs, heart, and hematopoietic tissues. Then, the sections were fixed in methanol for 10 min, and WSSV viral antigens were detected by IIF. WSSV-infected cells in the gills, hepatopancreas, lymphoid organ, coelomosac, heart, and hematopoietic tissues were counted in six randomly selected fields and expressed as the number of WSSV-infected cells per square millimeter. WSSV-infected and uninfected cells were counted randomly in six fields of hemocytes, bladder epithelial cells, and cuticular epithelial cells of the body, head, and hindgut and expressed as a percentage of infected cells.

#### Detection of WSSV viral antigens by IIF.

Viral antigen-positive cells in WSSV-infected shrimp were detected by IIF as described by ref. 29. Briefly, cephalothoraxes of dead, moribund, and killed shrimp were collected, embedded in 2% methylcellulose, and frozen at −20 °C. Cryosections (6 µm) were made and fixed for 10 min in 100% methanol at −20 °C. The sections were washed three times in PBS (5 min each) and incubated in 200 µL of monoclonal antibody w29 (directed against WSSV viral protein VP28 1:100 in PBS) at 37 °C for 1 h. Then, samples were washed three times in PBS (5 min each), and incubated with fluorescein isothiocyanate-labeled goat anti-mouse IgG (F-2761, Molecular Probes) for 1 h at 37 °C. Finally, the samples were washed twice with PBS, rinsed once in deionized water, and mounted with a solution of glycerine and 1,4-diaza-bicyclo-octane (ACROS Organics). Sections were analyzed with a confocal fluorescence microscope.

#### Statistical analysis.

Determination of viral infectivity (SID_50_) and bacterial lethal titers (LD_50_) was done based on the method of Reed and Muench (34).

### Morphology of the Antennal Gland.

Early premolt *P. vannamei* shrimp (MBW 9.13 ± 0.92 g) from the ARC were screened according to ref. 35 and brought to the Laboratory for Light and Electron Microscopy, Department of Morphology, Ghent University. Shrimp were humanely killed on ice, and cephalothoraxes were dissected and fixed in a solution of paraformaldehyde and picric acid (85 mL of 2% paraformaldehyde and 15 mL of saturated picric acid, pH 7.4) at 37 °C for 12 h. The cephalothoraxes were embedded in paraffin. Ten-micrometer-thick cross-sections (100 sections per mm) were made, and a hematoxylin−eosin staining was performed. Using AMIRA v6.0, the slides were then reconstructed to obtain a 3D image of the antennal gland. For the in vivo study of the antennal gland, we used µMRI; 15 shrimp were screened at different molting stages. Individual shrimp were transported to the Infinity laboratory, Ghent University. Magnetic resonance images were acquired on a 7-T small-animal MRI (Bruker BioSpin PharmaScan 70/16) using a transmit/receive volume coil with 40 mm inner diameter. A T2-weighted TurboRARE sequence was acquired using the following settings: repetition time 6,105 ms, echo time 37 ms, in-plane resolution 109 µm, 50 contiguous transverse slices of 600 µm, and a field-of-view of 30 × 25 mm, resulting in a total acquisition time of 17 min. Before commencing the MRI procedure, the shrimp were introduced into a modified falcon tube (50 mL) for fixation. The falcon tube was surrounded by a spiral of PVC tubing. To keep the shrimp immobile, the ambient temperature inside the falcon tube was reduced to 4 °C, by running ice-cold water through the tubing using a small electrical pump. Further immobilization was performed using small pieces of flexible foam. Magnetic resonance images were acquired using a T2-weighted sequence. Using InVesalius software, the area of the antennal gland at the height of the hepatopancreas was calculated following manual labeling and then put into ratio with the area of the hepatopancreas as a reference. The results were statistically analyzed using R (ANOVA and Pairwise *t* test with Bonferroni correction).

### Natural Infection of the Nephrocomplex.

#### Nephropore dynamics.

##### Determination of nephropore opening pressure in an ex vivo model.

**Fig. 7.**
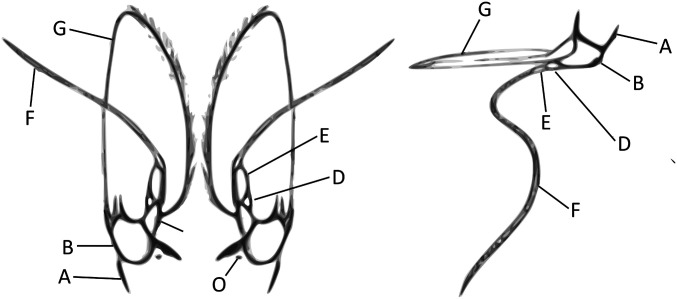
External morphology of the second segment (the antennal appendix) of *P. vannamei*. (*Left*) Ventral view and (*Right*) lateral view. A, coxipodite or coxicerite; B, basipodite or basicerite; C, ischiocerite; D, merocerite; E, carpocerite; F, flagellum; G, scaphocerite of exopodite of the second antenna; O: NP; A + B + C + D + E, pediculum of the antenna; A + B, protopodite of the second antenna; C + D + E, endopodite of the second antenna.

For the analysis of the urinal flow through the nephropore, an ex vivo model was set up to determine the pressure needed to open the nephropore valve and let urine flow from inside the shrimp to the outer world. Shrimp (MBW 15 g ± 1.02 g) of all molting stages, originating from the ARC, were humanely killed. The antennal appendix of each shrimp was carefully segregated from the rest of the shrimp body (Fig. 7). All inside tissues of the antennal appendix were removed with great care under a stereomicroscope (Bausch and Lomb), leaving only the exterior shell, that is, the cuticula with the nephropore. Next, the sample was mounted on a 50-mL plungerless syringe (Terumo Europe N.V.) such that the nephropore was situated in the middle of the tip opening of the syringe, with the inside of the cuticula facing the lumen of the syringe. A first fixation was achieved with superglue (Pattex, Henkel), and, after 5 min, the appendix was embedded into TEC7 (Novatech International) without obstructing the nephropore. The construct was left to dry for 3 h. To measure the pressure needed to evacuate fluid from inside of the shrimp to the outside, the syringe was filled with water until water leaked out of the nephropore. The height of the water was measured, and the pressure was calculated using the formula *P* = *ρ* × *g* × *h*, where *P* equals pressure (in Pascal), *ρ* equals the density of the liquid (1000 kg/m^3^), *g* equals the acceleration of gravity (9.81 m/s^2^), and *h* equals the leaking height of the fluid in the syringe. To determine the flow from outside to inside of the shrimp, the whole procedure was repeated with one difference: The nephropore was now not placed on the Luer tip facing up, but facing down, thus simulating possible influx of water in the shrimp.

##### Determination of fluid and particle influx in an ex vivo model after simulated urination.

The base of a plastic cryotube was cut off at 1 cm height, and a hole was made in the bottom. Through this hole, a 1-mL syringe without plunger was introduced and fixed using superglue (Pattex, Henkel). Next, the base of the cryotube was filled with TEC7 (Novatech International), leaving the aperture of the syringe uncovered by the substance. The glue was left to dry overnight. The antennal appendix, on which the nephropore is situated (Fig. 7), was removed from the shrimp as described in *Determination of nephropore opening pressure in an ex vivo model* and placed on top of the syringe opening and fixed with superglue, the inside of the cuticula shell facing the syringe. When dry, a mixture of TEC7 and a neoprene-based Bison glue (Bison International BV) was used to engulf the base of the antennal appendix (coxipodite), ensuring a watertight seam between the dried TEC7 and the cuticula. The glue was left to dry in 35‰ saline water overnight so the cuticula did not dry out. Next, only the antennal protopodite was kept, while the distal parts (the second antenna [ischiocerite, merocerite, carpocerite, and flagellum] and the scaphocerite) were removed. The resulting holes were filled with a mixture of TEC7 and neoprene-based Bison glue to ensure water tightness (50/50). Again, the glue was left to dry in 35‰ saline water overnight. The entirety was subsequently enrobed with a layer of superglue, leaving only the nephropore exposed. Functionality tests using water and a plunger proved that the valve-like mechanic of the nephropore was not altered after this procedure nor was the construct leaking water through the seams.

Afterward, the syringe was filled with 0.5 mL of DNA/RNase-free water, and the plunger was reintroduced. The nephropore was then submerged in 100 mL of DNA/RNase-free water containing 1- and 0.2-µm fluorescent beads (Distrilab) at a concentration of 10^5^ mL^−1^ each. The contents of the syringe were quickly pushed into the solution, simulating urination, followed by a short and quick pull of the plunger, mimicking the vacuum effect inside the cephalothorax following urine evacuation. The devise was removed from the mixture and rinsed with running DNA/RNase-free water and dried. The plunger was then pushed again with the nephropore above a microscope slide. A coverslip was mounted with nail polish, and the presence of particles was examined under a confocal fluorescent microscope (Leica).

#### Immersion of shrimp in water with WSSV during drop in salinity.

Twenty-five shrimp of ±35 g from the ARC were screened for molting stage C and brought to the Laboratory of Virology, Ghent University. After 1 wk of acclimatization, five animals were transferred to a single 10-L PVC tank with a partition in the middle (19). In the first half of the tank, 1 L of artificial seawater (35‰ salinity) was added and, in the other, 1 L of 5‰ artificial seawater. The shrimp started the experiment in the 35‰ half and, after 1 d of acclimatization, the animals were gently flipped to the 5‰ part of the tank. At the time of the flipping, 10^5.5^ SID_50_ mL^−1^ (1 L) of WSSV Thai-1 was present in this 5‰ half of the tank. After 5 h, the shrimp were gently flipped back to the 35‰ half of the tank. Every 12 h, urine and hemolymph were collected. Urine sampling was performed with a similar protocol of intrabladder inoculation; the tip (0.5 mm) of a 0.64 × 19 mm 26G Surflo-W catheter (Terumo) connected to a 1-mL syringe was introduced between the two valves of the nephropore. As soon as urine started to appear in the catheter due to capillary flow, a gentle vacuum was created using the syringe, extracting as much urine as possible without exposing the shrimp with unnecessary stress. Hemolymph was extracted using a method involving the introduction of a needle in the hemolymph sinus next to the heart. Next, DNA extraction (cador pathogen kit, Quigen) and subsequently qPCR were performed on both urine and hemolymph samples (36). The experiment was performed in triplicate. Two controls of five shrimp each were simultaneously performed: a control with a drop in salinity but without WSSV and a control without a drop in salinity but with WSSV.

## Supplementary Material

Supplementary File

## Data Availability

All study data are included in the article and *SI Appendix*.

## Change History

October 27, 2020: The license for this article has been updated.
