# Supplementary material for: The shrimp nephrocomplex serves as a major portal of pathogen entry and is involved in the molting process
Source: Proc Natl Acad Sci U S A. 2020 Oct 23;117(45):28374–83. doi: 10.1073/pnas.2013518117 (PMC7668069; doi:10.1073/pnas.2013518117)
Supplement: Supplementary File [file pnas.2013518117.sapp.pdf]

Supplementary Materials for

The shrimp nephrocomplex serves as a major portal of pathogen entry and is involved in the molting process

G.M.A. De Gryse[1]†, V.K. Thuong[1,2]†, B. Descamps[3], W. Van Den Broeck[4], C. Vanhove[3], P. Cornillie[4], P. Sorgeloos[5], P. Bossier[5], H.J. Nauwynck[1]*

Correspondence to: hans.nauwynck@ugent.be

This PDF file includes:

Supplementary movie S1
Supplementary data S1 to S6

www.pnas.org/cgi/doi/10.1073/pnas.2013518117

Movie S1.

Movie S1. 3D-rendering of the nephrocomplex, see link https://www.vpi.ugent.be/page-2/index.html

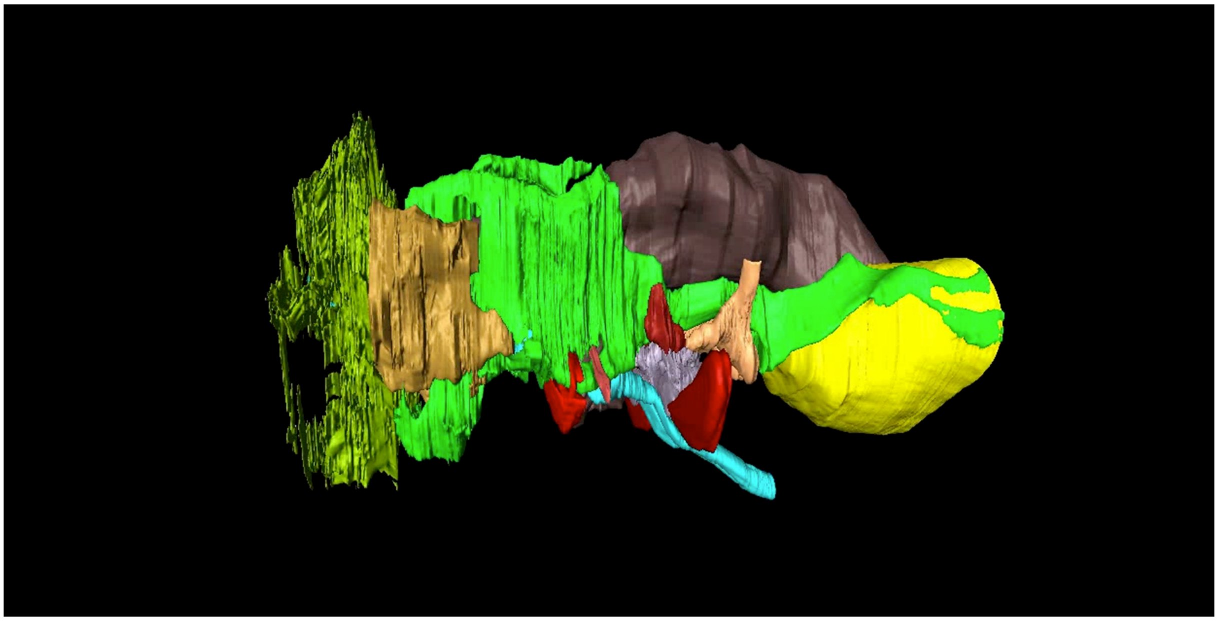

Data S1. Infectious virus titers of a WSSV stock upon intramuscular (im), peroral (po) and intrabladder inoculation in *P. vannamei*.

Experiment	Inoculation route	Dilution of WSSV stock	N	Number of dead animals at …. hpi									N IIF positive	Infectivity titer (SID_{50} ml^{-1})
				24	36	48	60	72	84	96	120	Total		
1	im	10^{-5}	5			3	1	1				5	5	$10^{8.67}$
		10^{-6}	5			2	2					4	4	
		10^{-7}	5									0	0	
	po	10^{0}	5			2						2	2	$10^{1.14}$
		10^{-1}	5									0	0	
		10^{-2}	5									0	0	
		10^{-3}	5									0	0	
	intrabladder	10^{-3}	5			2	2	1				5	5	$10^{6.97}$
		10^{-4}	5			1		2	1			4	4	
		10^{-5}	5				1	1				2	2	
		10^{-6}	5									0	0	
2	im	10^{-5}	5			3	2					5	5	$10^{8.67}$
		10^{-6}	5			1	2	1				4	4	
		10^{-7}	5									0	0	
	po	10^{0}	5			1	1					2	2	$10^{1.30}$
		10^{-1}	5					1				1	1	
		10^{-2}	5									0	0	
		10^{-3}	5									0	0	
	intrabladder	10^{-3}	5			3	2					5	5	$10^{6.60}$
		10^{-4}	5			1	1	1				3	3	
		10^{-5}	5				1					1	1	
		10^{-6}	5									0	0	
3	im	10^{-5}	5		3	1	1					5	5	$10^{8.80}$
		10^{-6}	5		2	1	1		1			5	5	
		10^{-7}	5									0	0	
	po	10^{0}	5				2					2	2	$10^{1.30}$
		10^{-1}	5					1				1	1	
		10^{-2}	5									0	0	
		10^{-3}	5									0	0	
	intrabladder	10^{-3}	5			2	2	1				5	5	$10^{7.30}$
		10^{-4}	5			2	1	1				4	4	
		10^{-5}	5				2		1			3	3	
		10^{-6}	5									0	0	

Data S2. Lethality titers of *Vibrio campbellii* upon intramuscular (im), peroral (po) and intrabladder inoculation in *P. vannamei.*

Experiment	Inoculation route	Dilution of *Vibrio*	N	Number of dead animals at different time points									Mortality (%)	Lethal titer (LD_{50} ml^{-1})
				0	6	12	18	24	30	36	120	Total		
1	im	10^{-2}	5		5							5	100	$10^{4.16}$
		10^{-3}	5		1	2						3	60	
		10^{-4}	5									0	0	
	po	10^{0}	5									0	0	-
		10^{-1}	5									0	0	
		10^{-2}	5									0	0	
	intrabladder	10^{0}	5			2	1					3	60	$10^{2.49}$
		10^{-1}	5			2						2	40	
		10^{-2}	5									0	0	
2	im	10^{-2}	5		3	2						5	100	$10^{4.37}$
		10^{-3}	5		2	2						4	80	
		10^{-4}	5									0	0	
	po	10^{0}	5									0	0	-
		10^{-1}	5									0	0	
		10^{-2}	5									0	0	
	intrabladder	10^{0}	5		2	1						3	60	$10^{2.49}$
		10^{-1}	5			2						2	40	
		10^{-2}	5									0	0	
3	im	10^{-2}	5		5							5	100	$10^{4.16}$
		10^{-3}	5		2	1						3	60	
		10^{-4}	5									0	0	
	po	10^{0}	5									0	0	-
		10^{-1}	5									0	0	
		10^{-2}	5									0	0	
	intrabladder	10^{0}	5		2	1						3	60	$10^{2.32}$
		10^{-1}	5			1						1	20	
		10^{-2}	5									0	0	

Supplementary data S3.

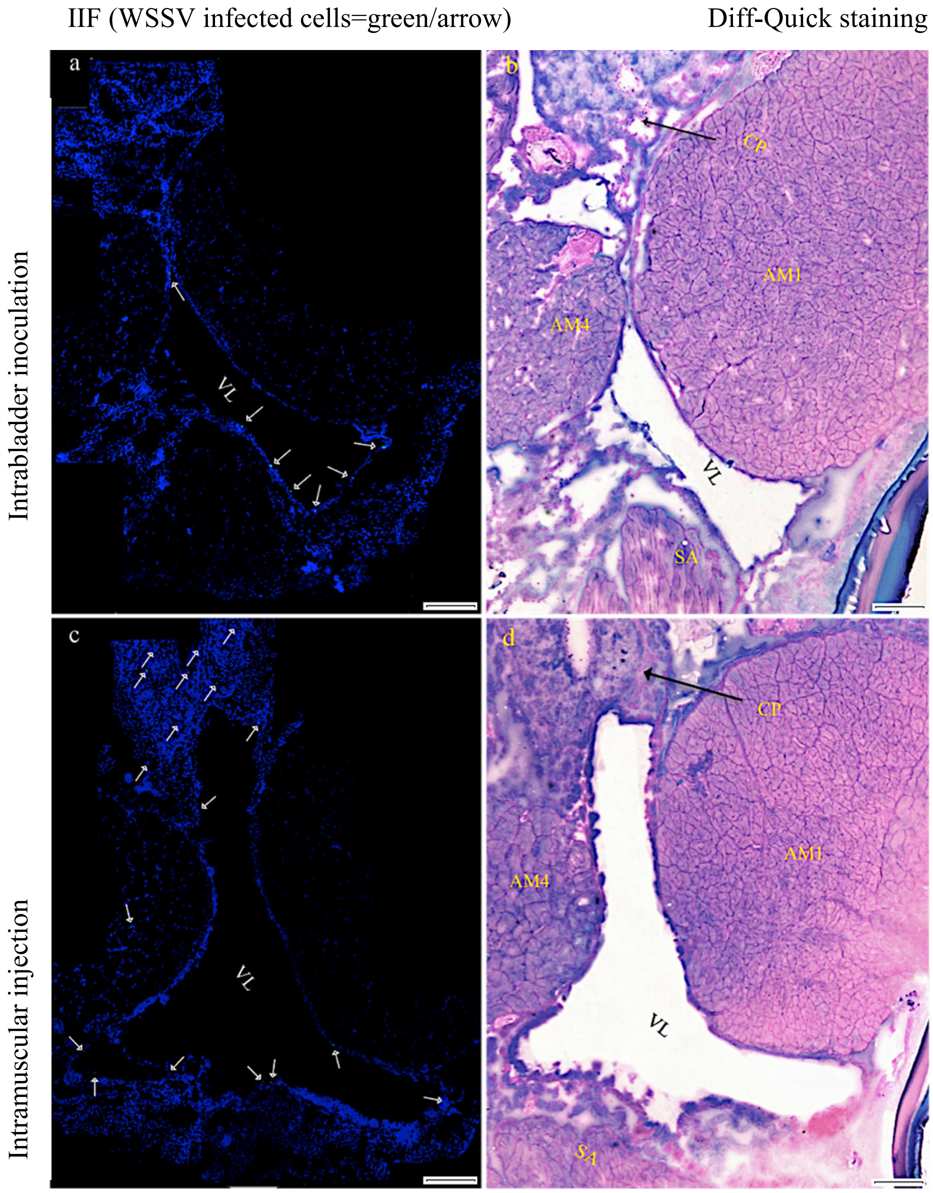

Supplementary Fig. S3a. Photomicrographs (PM) of nephrocomplex and surrounding areas of intramuscular and intrabladder WSSV-inoculated shrimp sampled at 18 hpi (a, b, c, d) and 24 hpi (e, f, g, h). Left panel: WSSV-infected cells were detected by IIF using a WSSV VP28-specific mouse monoclonal antibody and an FITC-conjugated goat anti-mouse IgG (green, see arrows); nuclei were visualized with Hoechst (blue). Right panel: for a correct orientation, cryosections were stained using Diff-Quick staining. PM a & b show the first WSSV-infected epithelial cells in the ventral bladder (VL) at 18 h after intrabladder inoculation; 6 hours later (e & f), WSSV positive cells were found in the ventral bladder, compact glandular compartment (CP) and muscles surrounding the nephrocomplex. PM c, d, g, h show WSSV-infected epithelial cells in the ventral bladder, compact glandular part and surrounding areas of the nephrocomplex at 18 and 24 h after intramuscular inoculation. AM1: adductor muscle of first antennal segment, AM4: adductor muscle of fourth antennal segment, SA: scaphocerite adductor muscle. Bar = 200 µm.

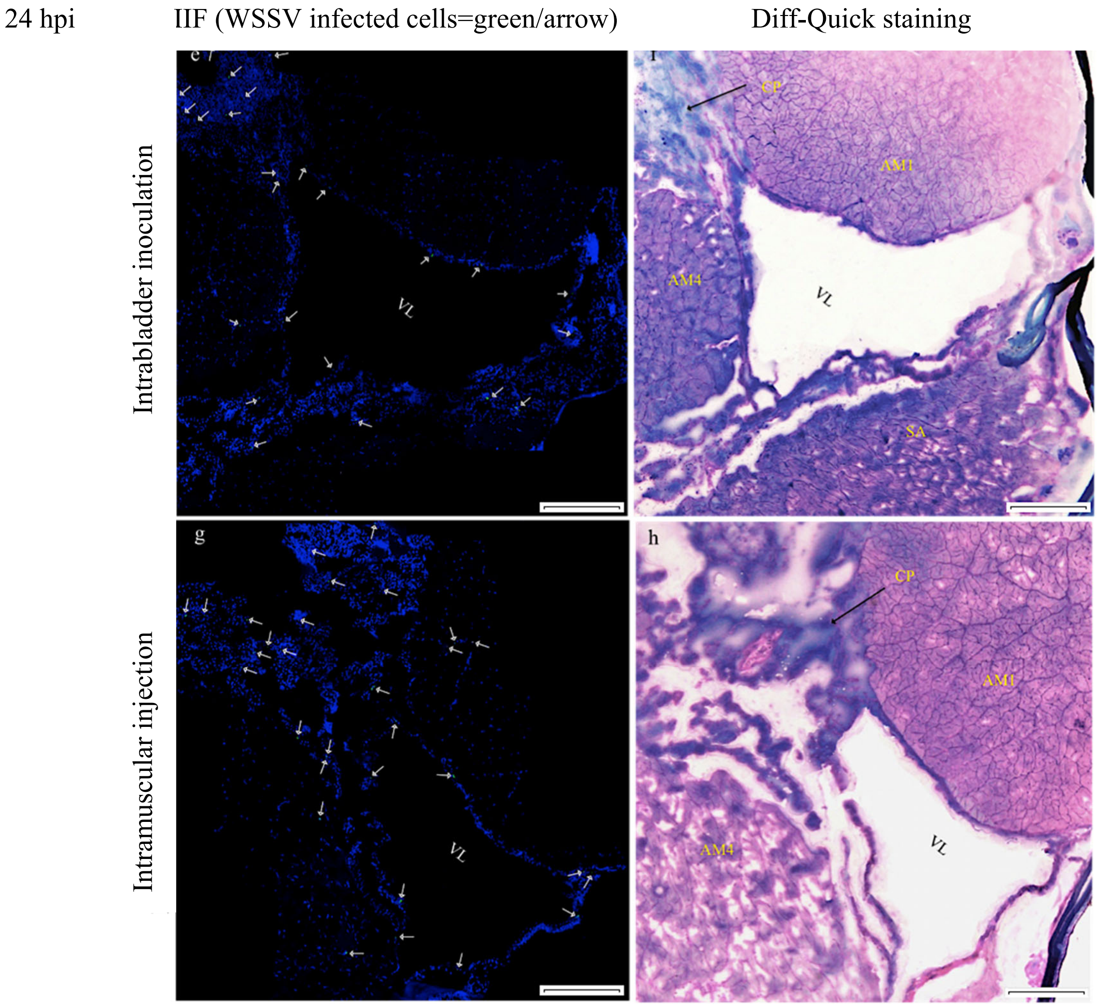

Supplementary Fig. S3b. Photomicrographs (PM) of nephrocomplex and surrounding areas of intramuscular and intrabladder WSSV-inoculated shrimp sampled at 18 hpi (a, b, c, d) and 24 hpi (e, f, g, h). Left panel: WSSV-infected cells were detected by indirect immunofluorescence using a WSSV VP28-specific mouse monoclonal antibody and an FITC-conjugated goat anti-mouse IgG (green, see arrows); nuclei were visualized with Hoechst (blue). Right panel: for a correct orientation, cryosections were stained using Diff-Quick staining. PM a & b show the first WSSV-infected epithelial cells in the ventral bladder (VL) at 18 h after intrabladder inoculation; 6 hours later (e & f), WSSV positive cells were found in the ventral bladder, compact glandular compartment (CP) and muscles surrounding the nephrocomplex. PM c, d, g, h show WSSV-infected epithelial cells in the ventral bladder, compact glandular part and surrounding areas of the nephrocomplex at 18 and 24 h after intramuscular inoculation. AM1: adductor muscle of first antennal segment, AM4: adductor muscle of fourth antennal segment, SA: scaphocerite adductor muscle. Bar = 200 µm.

Data S4. Quantification of infected cells in various organs of *P. vannamei* shrimp inoculated with WSSV via intramuscular (im) and intrabladder inoculation.

		Quantity of WSSV-infected cells in											
		Nephrocomplex								Cuticular epithelium			
Inoculation route	Time (hpi)	Ventral bladder epithelium (%)	Labyrinth (mm^{-2})	Coelomosac (mm^{-2})	Hematopoietic tissue	Hepato-pancreas (mm^{-2})	Heart (mm^{-2})	Lymphoid organ (mm^{-2})	Gills (mm^{-2})	Head cuticular epithelium (%)	Body cuticular epithelium (%)	Hindgut cuticular epithelium (%)	Hemolymph (%)
im	0	0	0	0	0	0	0	0	0	0	0	0	0
	6	0	0	0	0	0	0	0	0	0	0	0	0
	12	0	0	0	0	0	0	0	0	0	0	0	0
	18	0.8±0.5	7±10	43±29	25±16	36±18	13±13	19±10	41±28	0.3±0.2	0.6±0.5	0.5±0.4	0
	24	2.0±1.2	66±24	93±34	81±27	157±50	68±37	62±20	249±75	1.8±0.6	2.7±1.5	3.5±2.4	0.4±0.3
	36	8.4±3.7	341±96	739±93	593±347	336±107	304±129	226±81	847±160	20.4±7.9	16.4±3.1	13.9±6.4	0.8±0.5
	48	13.3±4.5	914±301	2,057±274	1,186±354	795±203	504±270	981±221	2,037±207	22.9±7.6	35.9±7.1	32.9±6.3	1.5±0.4
Intra-bladder	0	0	0	0	0	0	0	0	0	0	0	0	0
	6	0	0	0	0	0	0	0	0	0	0	0	0
	12	0	0	0	0	0	0	0	0	0	0	0	0
	18	0.8±0.4	0	0	0	0	0	0	0	0	0	0	0
	24	1.6±0.9	8±7	84±27	23±11	15±12	12±9	11±7	16±10	0.3±0.2	0.7±0.4	0.3±0.2	0.1±0.1
	36	5.9±1.9	138±35	561±114	187±121	233±48	205±72	179±39	407±179	11.6±4.0	6.0±2.8	5.6±1.9	0.6±0.4
	48	6.7±2.6	392±75	1,980±331	573±212	405±166	408±158	420±151	1,861±205	17.6±4.5	24.7±6.2	22.6±6.1	1.0±0.4

Data S5. WSSV genome copies in urine and hemolymph after immersion inoculation ($10^{5.5}$ SID_{50} ml^{-1}) during a 5 h drop in salinity from 35 g L^{-1} to 5 g L^{-1} (three repeats and two controls).

Repeat 1

	Genome copies µl^{-1}									
	Shrimp 1		Shrimp 2		Shrimp 3		Shrimp 4		Shrimp 5	
Collection time (hpi)	Urine	Hemolymph	Urine	Hemolymph	Urine	Hemolymph	Urine	Hemolymph	Urine	Hemolymph
0	0	0	0	0	0	0	0	0	0	0
12	18	0	3	0	23	20	16	0	20	0
24	58	2	7	52	12	144	23	1	60	346
36	1,050	1,583	26	1,567	116	70,945	130	729	121	686,174
48	dead		dead		dead		dead		dead	

Repeat 2

	Genome copies µl^{-1}									
	Shrimp 1		Shrimp 2		Shrimp 3		Shrimp 4		Shrimp 5	
Collection time (hpi)	Urine	Hemolymph	Urine	Hemolymph	Urine	Hemolymph	Urine	Hemolymph	Urine	Hemolymph
0	0	0	0	0	0	0	0	0	0	0
12	78	0	0	0	11	0	1	66	4	0
24	dead	747a	1	182	Dead	108a	3,636	8,695,294	0	787
36			0	458,755			5,366	1,756,139	847	7,550
48			dead	733,907a			dead		dead	2,366a

[a] Postmortem sampling

Repeat 3

	Genome copies µl^{-1}									
	Shrimp 1		Shrimp 2		Shrimp 3		Shrimp 4		Shrimp 5	
Collection time (hpi)	Urine	Hemolymph	Urine	Hemolymph	Urine	Hemolymph	Urine	Hemolymph	Urine	Hemolymph
0	0	0	0	0	0	0	0	0	0	0
12	3	0	10	0	11	4	1	9	4	0
24	4,300	538	139	184	236	317	4,360	733,907	36	1,213
36	2,323	236,567	90	301,523	64,320	4,280,702	dead		870	35,010
48	dead		dead		dead				dead	

Control 1 (with a drop in salinity but without WSSV; all shrimp survived)

	Genome copies/µl									
	Shrimp 1		Shrimp 2		Shrimp 3		Shrimp 4		Shrimp 5	
Collection time (hpi)	Urine	Hemolymph	Urine	Hemolymph	Urine	Hemo-lymph	Urine	Hemolymph	Urine	Hemolymph
0	0	0	0	0	0	0	0	0	0	0
12	0	0	0	0	0	0	0	0	0	0
24	0	0	0	0	0	0	0	0	0	0
36	0	0	0	0	0	0	0	0	0	0
48	0	0	0	0	0	0	0	0	0	0

Control 2 (without drop in salinity but with WSSV; all shrimp survived)

	Genome copies/µl									
	Shrimp 1		Shrimp 2		Shrimp 3		Shrimp 4		Shrimp 5	
Collection time (hpi)	Urine	Hemolymph	Urine	Hemolymph	Urine	Hemolymph	Urine	Hemolymph	Urine	Hemolymph
0	0	0	0	0	0	0	0	0	0	0
12	0	0	0	0	0	0	0	0	0	0
24	0	0	0	0	0	0	0	0	0	0
36	0	0	0	0	0	0	0	0	0	0
48	0	0	0	0	0	0	0	0	0	0

Data S6. Pressure needed to open the nephropore from inside the shrimp at different molting stages

Molting stage	#	Height water column (cm)	Average	Pressure (Pa)	Pressure (bar)
A	1	29		284490	2.84
	2	26	28.3	255060	2.55
	3	30		294300	2.94
B	1	30		294300	2.94
	2	26	28.3	255060	2.55
	3	29		284490	2.84
C	1	29		284490	2.84
	2	27	28.1	264870	2.65
	3	30		294300	2.94
D1	1	28		274680	2.75
	2	30	29	294300	2.94
	3	29		284490	2.84
D2	1	30		294300	2.94
	2	29	29.3	284490	2.84
	3	29		284490	2.84
